# Direct Observation of Discharge Phenomena in Vibration-Assisted Micro EDM of Array Structures

**DOI:** 10.3390/mi13081286

**Published:** 2022-08-10

**Authors:** Gero Esser, Jiwang Yan

**Affiliations:** Department of Mechanical Engineering, Faculty of Science and Technology, Keio University, Yokohama 223-8522, Japan

**Keywords:** micro electrical discharge machining, vibration assisted electrical discharge machining (EDM), high-speed camera, array electrode, structured surface

## Abstract

The batch mode electrical discharge machining (EDM) method has been developed to improve the throughput and accuracy in fabricating array structures, but the process suffers from insufficient debris removal caused by the complex electrode geometry. Tool vibration has been used to improve flushing conditions, but to date the underlying mechanism of the tool vibration on the micro EDM of array structures remains unclear. This study aimed to investigate the effect of tool vibration on the machining process by direct observation of the discharge phenomena in the discharge gap by using a high-speed camera. Micro EDM experiments using 9 and 25 array electrodes were performed, and the effect of tool vibration on the discharge uniformity and tool wear was evaluated. It was found that tool vibration improved the uniformity of the discharge distribution, increased the machining efficiency, and suppressed the tool wear. The discharges occurred in periodic intervals, and the intensity increased with the amplitude of tool vibration. The results of this study indicate that the vibration parameters determine the discharge period duration and intensity to achieve optimum stability and efficiency of the machining process.

## 1. Introduction

Electrical Discharge Machining (EDM) is a non-traditional machining method with outstanding performance for precision machining of hard and brittle materials [1]. The material removal is based on the thermal effect of electrical discharges in a narrow gap between the tool and workpiece electrode. The gap is filled with a dielectric fluid to enable controlled discharges and the transportation of removed material [2]. Accumulation of removed material as debris particles in the discharge gap is a significant problem in EDM. A higher debris concentration leads to a decreased dielectric breakdown strength, causing unstable machining and short-circuiting [3]. As a result, the machining time increases, and the machining quality can be reduced [4,5]. Uniform debris distribution and timely removal of particles from the gap are thought to be the main factors contributing to a stable machining process [6].

Micro array fabrication is a specific application of micro EDM that has gained increasing attention in recent years. Li et al. [7] used wire Electrical Discharge Grinding (WEDG) to fabricate micro electrodes for micro EDM drilling of arrays with 2 by 128 holes for inkjet printer nozzles. Li et al. [8] fabricated micro hole arrays for the micro embossing of lens arrays by micro EDM drilling. As the accuracy of the tool electrode determines the machining quality of the array, several studies have been carried out to improve the fabrication process and wear performance of micro electrodes for micro EDM drilling of arrays [9,10]. Debnath and Patowari [11] proposed wire EDM to fabricate arrays of rectangular micro pillars as a heat exchanger for small-scale electronic devices. However, the machining accuracy of the arrays was low, with a pillar thickness error of about 25 µm.

The serial process for micro EDM drilling single holes of the array can be parallelized by using array electrode tools. This batch mode machining method is a promising alternative to overcome the challenges of limited throughput and severe wear in the micro EDM drilling of arrays [12,13]. Yi et al. [13] demonstrated the machining of steel shadow masks by batch mode micro EDM with arrays of 4 by 4 rectangular electrodes to fabricate organic thin-film transistors. Zeng et al. [14] studied the performance of ultrasonic vibration-assisted micro EDM machining with arrays of 5 by 5 electrodes. Other applications of micro arrays include filters, fuel nozzles, semiconductor and electronics components, and biotechnology devices [9,12]. The fabrication process of array tools with complex geometry was addressed, and electrode fabrication by reverse EDM, wire EDM, lithography, electroplating, and molding (Lithographie, Galvanoformung, and Abformung (LIGA)), and micro milling was reported [15,16,17,18]. Chen [16] designed a multidirectional wire EDM machine tool and fabricated arrays of electrodes with 21 µm width and 700 µm length. Wang et al. [18] developed an integrated machine tool for on-machine micro milling of 3 by 3 rectangular array electrodes and micro EDM of cavities for improved machining accuracy. Sun et al. [19] fabricated array electrodes by reverse EDM and demonstrated that the effect of tool wear could be utilized to fabricate hole arrays with rectangular inlets and circular outlets.

One of the main issues in batch mode manufacturing of arrays is the difficult debris removal due to the complex shaped workpiece–electrode gap. Process instability and nonuniformity caused by debris accumulation is a major problem for array EDM. A variation of the hole diameter and depth across the array has been a common problem in several studies [12,14,20]. To minimize debris accumulation in the discharge gap, methods have been developed to improve debris flushing conditions. Tool rotation is often used for micro EDM drilling, but it is limited to tool geometries with cylindrical shapes and cannot be applied to array tools in batch mode machining [21]. As an alternative, vibration of the tool or workpiece was proposed to improve micro EDM machining of array structures and other non-cylindrical geometries [4]. Unlike other flushing methods, the vibration assistance is effective even when the gap is extremely small, which makes the tool vibration method suitable for micro EDM [22]. Several studies have found that tool vibration can increase the machining stability and reduce the machining time [23,24]. Tong et al. [6] observed an improved accuracy by workpiece vibration in the machining of micro gears. It has been demonstrated that vibration also affects tool wear and surface roughness [23,24,25]. However, the fundamental mechanism of vibration-assisted EDM and the effect of vibration frequency and amplitude on the machining process for micro EDM of array structures are not yet fully understood. In addition, optimization of the vibration conditions is necessary for industrial applications.

The extremely rapid character of the discharge phenomena in the small gap complicates the investigation of micro EDM mechanisms [2]. Many different physical/chemical processes such as electrical discharges, transfer of heat and material, and chemical reactions interact in the gap. This complexity intricates analytical and numerical modeling [26]. Kitamura et al. [2] developed a direct observation method of the discharge gap that has been used for the clarification of gap phenomena in EDM. The discharge gap of a rectangular electrode was studied. It was found that most of the gap was filled by bubbles, but discharges were mostly observed in the dielectric liquid [2]. In [27], the discharge locations in wire EDM were observed, and the authors suggested that a larger distance of consecutive discharges contributed to a better stability of the machining process. Yue et al. [28] demonstrated that the phenomena in the discharge plasma could be made visible by using bandpass filters or laser illumination. An influence of the gap flow field on the material removal was concluded from the observations. The discharge gap in vibration-assisted micro EDM using cylindrical electrodes was observed by Shitara et al. [24]. The position of discharge spots was evaluated, and the distribution of discharge locations was found to be more uniform with the vibration of the electrode. A so-called interrupted discharge phenomenon was observed where discharges only occurred during a short period of the electrode movement.

**Figure 1:**
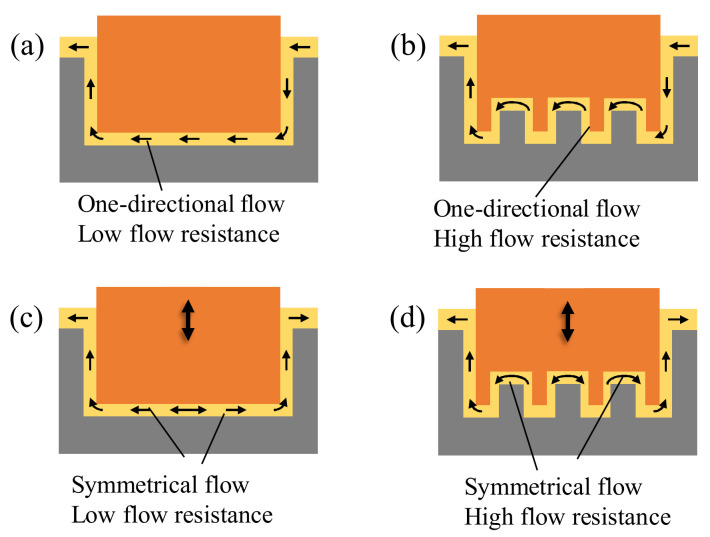
Effect of vibration and difference of the flow field (**a**) single electrode without vibration, (**b**) array electrode without vibration, (**c**) single electrode with vibration, (**d**) array electrode with vibration.

The schematic in Figure 1 illustrates the flow field in the gap in micro EDM of single and array electrodes with/without tool vibration. A higher flow resistance results from the array electrode geometry with a long, curved gap. This is associated with a more difficult machining without tool vibration. In general, the tool vibration generates a symmetrical fluid motion in the center region of the electrode end towards the side gap. In the gap of the array electrode, the flow is symmetrical in the center but one-directional towards the side areas. The effects of the difference in local flow direction and flow resistance on the non-uniformity of machined surface accuracy are unknown.

To date, most studies on the gap observation of EDM, especially vibration-assisted EDM, used simple tool geometries such as single rectangular or circular electrodes. Though the application of tool vibration shows its full potential in micro EDM of complex geometries, such as multiple-cavity arrays, the mechanisms underlying this specific situation have not been clarified.

In this study, the direct observation of the discharge gap is applied to micro EDM with array electrodes. The study aims to investigate the fundamental effects of tool vibration assistance by evaluating the distribution and time-dependent change of the discharges. The machining performance improvement mechanism will be examined, and the potential of vibration assistance for micro EDM of complex geometries will be demonstrated. The findings of the discharge phenomena and mechanisms will be useful for the development of optimal vibration conditions for high-uniformity micro fabrication of large-area array structures.

## 2. Materials and Methods

Micro EDM experiments were performed, and the discharges were recorded by direct gap observation. Image processing was applied to evaluate the discharge locations.

**Figure 2:**
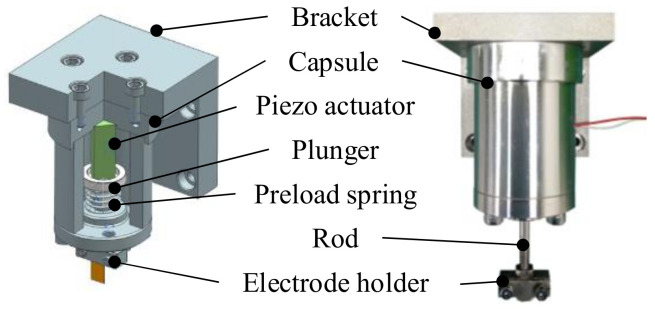
Cross-section and overview of the components of the vibration unit.

An actuator was required to generate the vibration motion of the tool electrode. A piezoelectric actuator was selected due to the fast response time and high acceleration. The design and verification process described in [29] was followed. The vibration unit illustrated in Figure 2 was designed using a piezoelectric transducer (AE0505D16F, Tokin Co., Sendai, Japan). The frequency and amplitude of the driving voltage were controlled to set the desired displacement. The axial and radial tool motion was analyzed by a laser displacement sensor (LK-H025, Keyence, Osaka, Japan), and low radial movement was confirmed. The vibration modes in Table 1 were selected for the experiments. Throughout this paper, we use the term amplitude for the peak-to-valley amplitude of the vibration motion.

**Figure 3:**
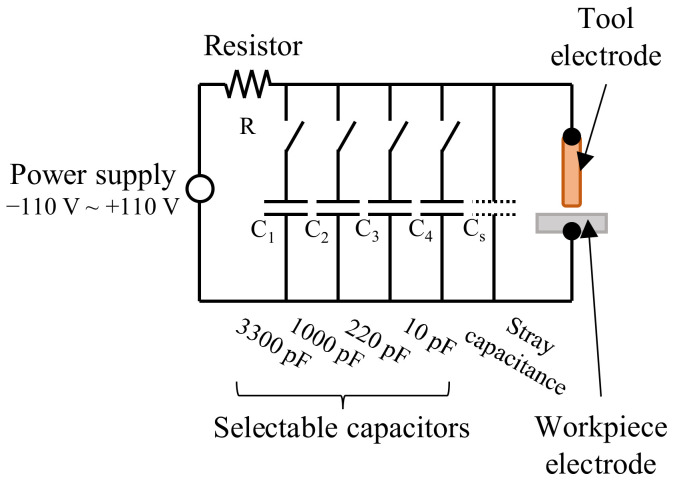
Schematic of the resistor–capacitor type EDM circuit.

A resistor-capacitance (RC) type EDM machine (MG-ED72, Panasonic, Kadoma, Japan) was used to conduct the experiments. As seen in Figure 3, the discharge energy was controlled by setting the voltage and capacitance of the EDM circuit. The experimental parameters of the machine are detailed in Table 2.

**Figure 4:**
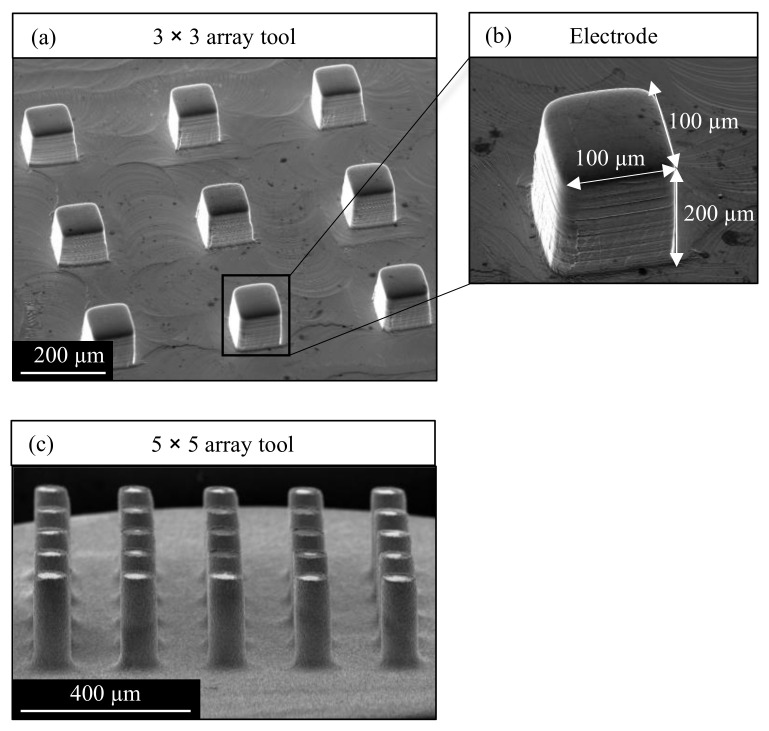
SEM image of the tool array electrodes used in this study. (**a**) Tool array of 9 rectangular electrodes (**b**) Detailed view of an electrode of the array (**c**) Tool array of 25 cylindrical electrodes.

Micro EDM experiments with arrays of 9 and 25 electrodes were performed, and the effect of tool vibration on the machining process was investigated. Figure 4a illustrates the tool array of 9 rectangular electrodes with a size of 100 µm by 100 µm and a height of 200 µm. The electrodes were fabricated from a copper rod of 2 mm diameter by micro milling as it is economical and less time-consuming than other manufacturing methods while achieving sufficient accuracy for the observation experiments. The top surface of the array was hand polished to remove burrs from micro milling and to increase the surface reflectivity of the electrodes to ease the high-speed camera observation. Before the experiments, the tool and workpiece were cleaned in an ultrasonic bath with EDM oil. Tool arrays of 25 cylindrical electrodes are illustrated in Figure 4c. The arrays were fabricated from copper material by reverse EDM to achieve higher accuracy and smaller spacing of the electrodes. An electrode diameter of 80 µm, spacing of 200 µm, and electrode length of 200 µm was achieved.

A workpiece material with sufficient electrical conductivity for EDM processing and transparency to enable observation of the gap phenomena was required. Semiconductor materials such as SiC and Ga_2_O_3_ have been used in previous studies [30]. Despite lower machinability [30], SiC was selected in this work due to its lower cost and better availability. Rectangular pieces of the 4H-SiC wafer (10 mm × 10 mm × 365.7 μm) were used as workpiece electrodes. 4H-SiC is a polar material due to the bilayer stacking sequence of Silicon and Carbon [31,32]. The machining was performed on the Carbon face of the SiC material.

**Figure 5:**
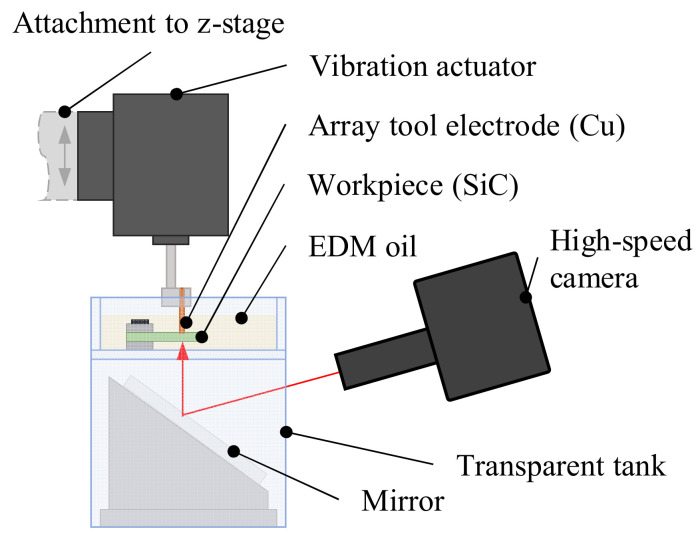
Schematic of the setup for the direct observation of the discharge gap.

**Figure 6:**
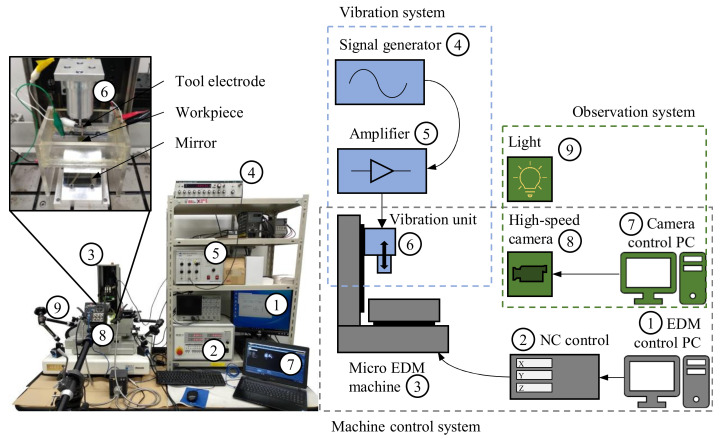
Block diagram representation and photo of the experiment setup.

The observation was conducted by a high-speed camera (FASTCAM Mini AX50, Photron Ltd., Tokyo, Japan). The settings of the high-speed camera are shown in Table 3. The experiment setup is illustrated in Figure 5 and was based on previous studies on direct gap observation [2,24,30]. As seen from the schematic in Figure 6, the vibration unit was connected to a signal generator and amplifier and mounted to the z-axis of the micro EDM machine. The tool electrode was attached to the vibration unit. The SiC workpiece was installed in an acrylic tank and submerged in the dielectric fluid. A mirror was placed on an inclined stand beneath the tank. The high-speed camera and high luminance lights were set up in front of the micro EDM machine. The discharge gap was observed through the transparent SiC workpiece from below via the mirror.

Die sinking EDM was performed, and the machining progress was tracked. The gap observation settings were optimized to show only the discharges and minimize the interference of bubbles, debris, and other effects. The video recording was started after the electrode reached a machining depth of 50 µm. Machining showed significant differences at this depth, and suitable evaluation results were obtained due to low interference of reflections on the recorded videos.

After the experiments, the gap videos and the machined cavities were analyzed. An image-processing script was developed in MATLAB to evaluate the discharges in the recorded videos. The machined surface of the SiC samples was scanned by a non-contact laser profilometer (NH-3SPs, Mitaka Kohki Co., Tokyo, Japan) and inspected by SEM (Inspect F50, FEI Co., Hillsboro, OR, USA).

## 3. Results and Discussion

### 3.1. Discharge Distribution States

**Figure 7:**
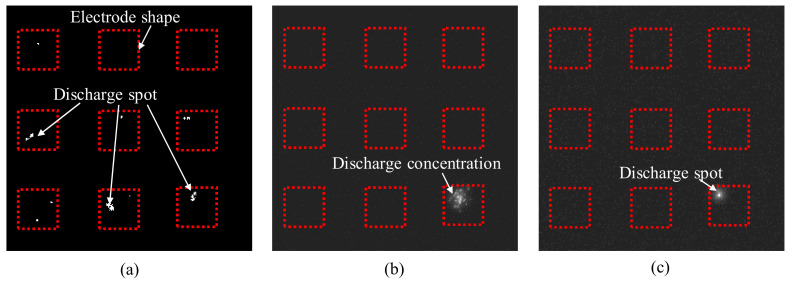
Different discharge states observed at 50 Hz and 5 µm vibration with the EDM parameters specified in Table 2: (**a**) unform distribution, (**b**) concentration at a single electrode, (**c**) single discharge spot.

In contrast to machining without vibration, where discharges occurred randomly, tool vibration led to a periodic discharge. In regular intervals, the discharges were stopped or only observed sporadically. In the experiments, three different discharge states were observed through the videos. Generally, multiple discharge spots were distributed over several electrodes of the array. Figure 7a shows a single frame recorded during this regular machining state. Occasionally discharges occurred concentrated on a single electrode in a high number. This discharge concentration is visible as a bright area in Figure 7b. The discharge concentration usually occurred at the end of the regular discharge period. Intense discharge continued for an extended time in the vibration cycle, even after regular discharge on the other electrodes was stopped. The discharge concentration phenomenon often appeared repeatedly over multiple vibration cycles. Infrequently, single bright spots, as shown in Figure 7c, that remained for several consecutive frames were observed. In most cases, this phenomenon followed a discharge concentration. The bright spot was assumed to be caused by arcing or short-circuiting when debris particles formed a bridge between the tool and the workpiece. The bright spot phenomenon often observed after the discharge concentration in the videos provides additional support for the findings of Shitara et al. [24] that discharge concentration might be a precursor to short-circuiting.

### 3.2. Effect of Vibration on Discharge Distribution

**Figure 8:**
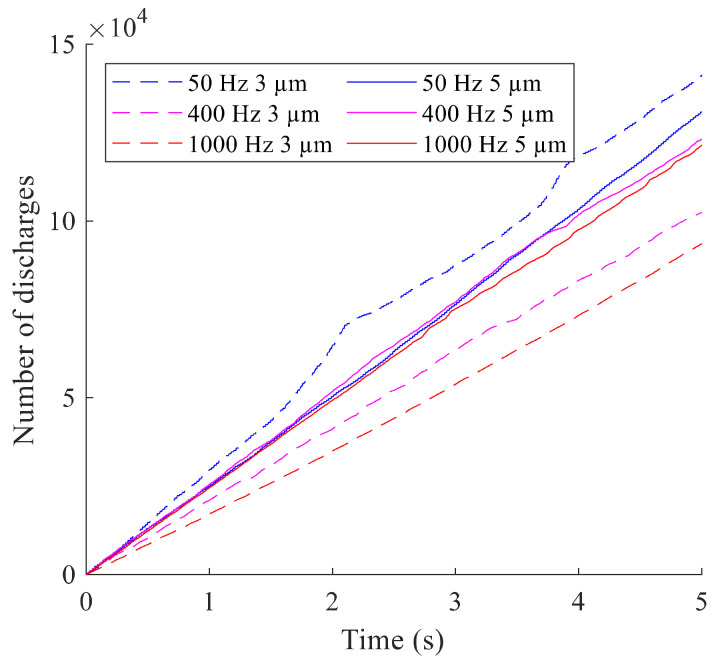
Number of discharges over the recording time.

The video was evaluated by thresholding the grayscale video frames in MATLAB. The location and number of the bright discharge spots in each frame were detected. The number of discharges with tool vibration is illustrated in Figure 8. The highest discharge rate was recorded for vibration at 50 Hz and 3 µm, but irregularities were seen twice. All other vibration modes showed good linearity. The discharge rates at 50 Hz, 400 Hz, and 1000 Hz with an amplitude of 5 µm were higher than the discharge rates at 400 Hz and 1000 Hz with 3 µm amplitude.

**Figure 9:**
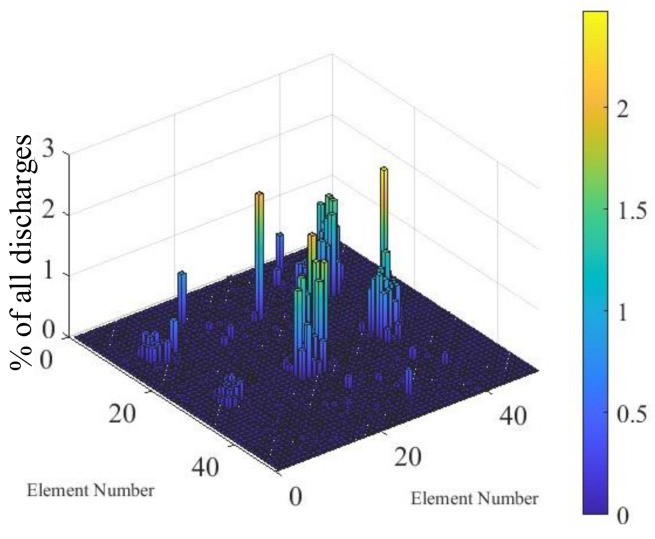
Distribution of discharges without tool vibration and the EDM parameters specified in Table 2.

The frames were partitioned in a grid of 50 by 50 elements, and the number of discharge spots in each element was counted. Without tool vibration, only a small number of discharges were observed, concentrated on a few electrodes of the array. This resulted in the highly non-uniform distribution, as shown in Figure 9.

**Figure 10:**
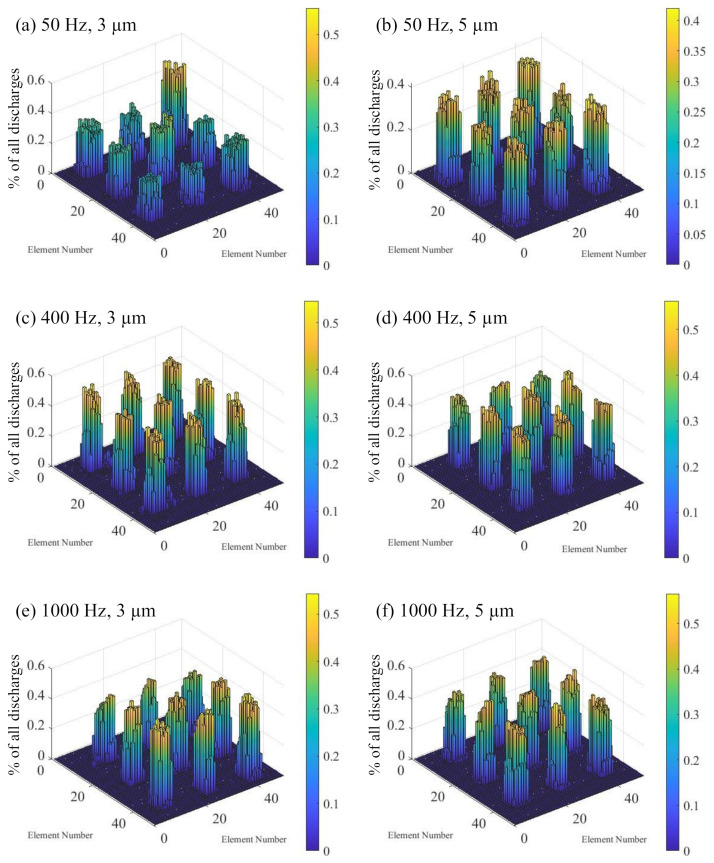
Distribution of discharges with tool vibration and the EDM parameters specified in Table 2. The vibration frequencies and amplitudes are: (**a**) 50 Hz, 3 µm (**b**) 50 Hz, 5 µm (**c**) 400 Hz, 3 µm (**d**) 400 Hz, 5 µm (**e**) 1000 Hz, 3 µm (**f**) 1000 Hz, 5 µm.

Such non-uniform distribution was also observed during vibration-assisted EDM at 50 Hz and 3 µm amplitude. As illustrated in Figure 10a, a much higher number of discharges was observed on the top-right electrode, while the number of discharges on the center electrode was slightly increased. The gap observation video showed that this was a result of discharge concentration. The discharge concentration was observed twice on the top-right and once on the center electrode during the recorded timespan.

The distribution of discharge spots for the vibration mode of 50 Hz and 5 µm amplitude is given in Figure 10b. The uniformity of the discharge distribution was significantly increased. This indicates higher stability of the machining process. The recorded video showed that discharge concentration occurred once on the bottom-right electrode. However, the concentration continued only for a few vibration cycles and did not influence the discharge distribution. Subsequently, the bright spot phenomenon was observed in the location where the discharge concentration occurred.

The discharge distributions at 400 Hz and 1000 Hz with both 3 µm and 5 µm amplitude are illustrated in Figure 10c–f. Machining at these vibration parameters showed good uniformity. A correlation of the discharge uniformity to the frequency or amplitude of the vibration was not identified for these parameters. Compared to the 50 Hz and 5 µm vibration result, the distribution was slightly less uniform. The reasons for this can be found in the higher variation of the discharge numbers in the elements on individual electrode positions. An uneven electrode surface may have resulted in regions with more discharges. Elevations could have been caused by the adhesion of debris or deposited carbon from the EDM oil. A gradual decrease of discharges across the array was observed in some cases, such as at 400 Hz and 5 µm. A higher concentration of debris may have increased the discharge number in one electrode area. It is also possible that some discharges were not visible due to a brightness gradient of the lighting in the gap.

### 3.3. Effect of Vibration on the Discharge Period

**Figure 11:**
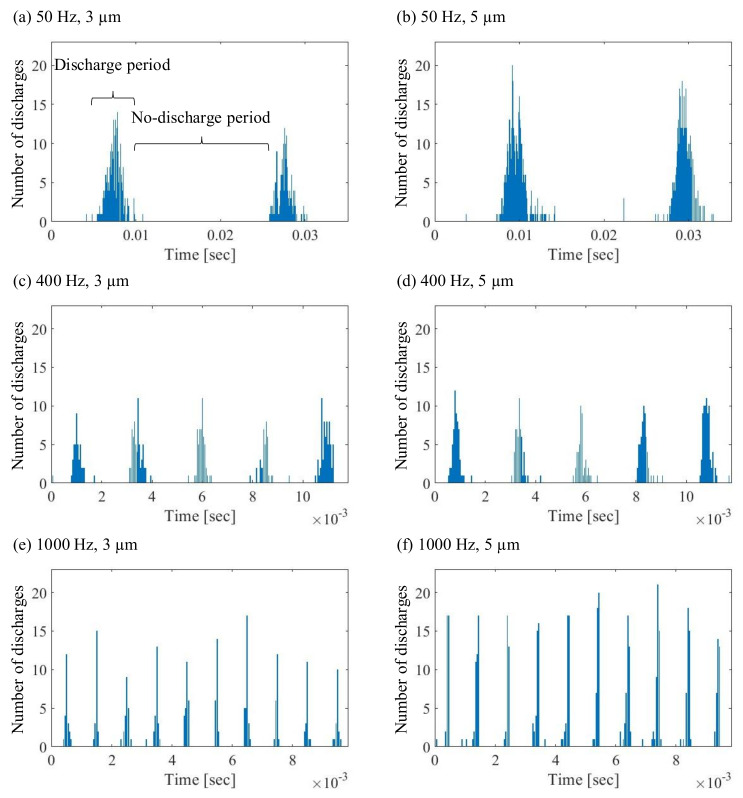
Discharge periods with the parameters specified in Table 2. The vibration frequencies and amplitudes are: (**a**) 50 Hz, 3 µm (**b**) 50 Hz, 5 µm (**c**) 400 Hz, 3 µm (**d**) 400 Hz, 5 µm (**e**) 1000 Hz, 3 µm (**f**) 1000 Hz, 5 µm.

The duration and intensity of the periods with/without discharge were analyzed. The number of discharge spots detected per frame at an arbitrary position is displayed in Figure 11. With increasing frequency, the beginning and end of the discharge periods became more apparent as the number of stray discharges decreased. The duration and number of discharges of the discharge periods was evaluated, and the average discharge intensity over the recorded video is illustrated in Table 4. There was a significant correlation between the vibration amplitude and the intensity of the discharge rate. At all observed vibration frequencies, a higher rate of discharge spots during the discharge period was found when the vibration amplitude was increased from 3 µm to 5 µm.

**Figure 12:**
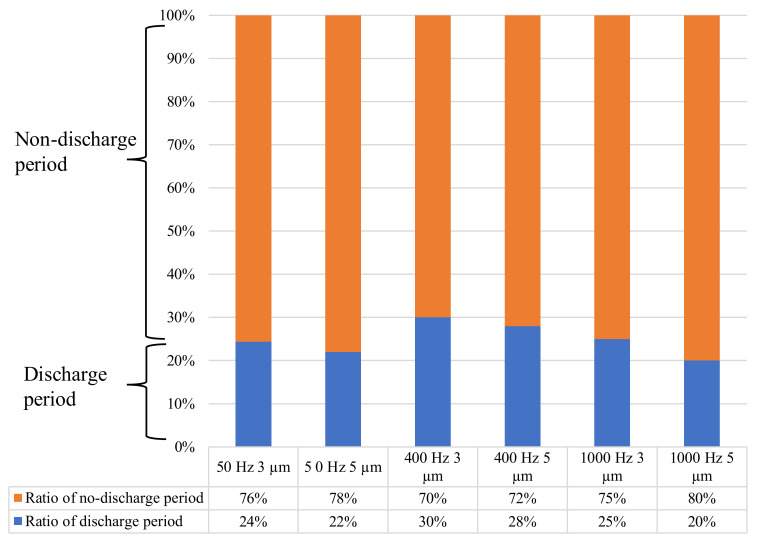
Ratio of the average discharge period and no-discharge period duration in the vibration cycle.

Figure 12 shows the ratio of the average discharge period duration to the average no-discharge period. It was found that an increase of both the vibration frequency and amplitude led to a shorter discharge period. This was attributed to the relation of the vibration parameters and the electrode velocity, as detailed in Equation (1). The electrode velocity, *v*, varies with the time, *t*. The vibration frequency and amplitude are denoted by *f* and $\hat{a}$, while *φ* is the phase shift.

(1) $$v\left(t)=\hat{a}\pi f\;\sin(2\pi f\;t+\varphi \right)$$

**Figure 13:**
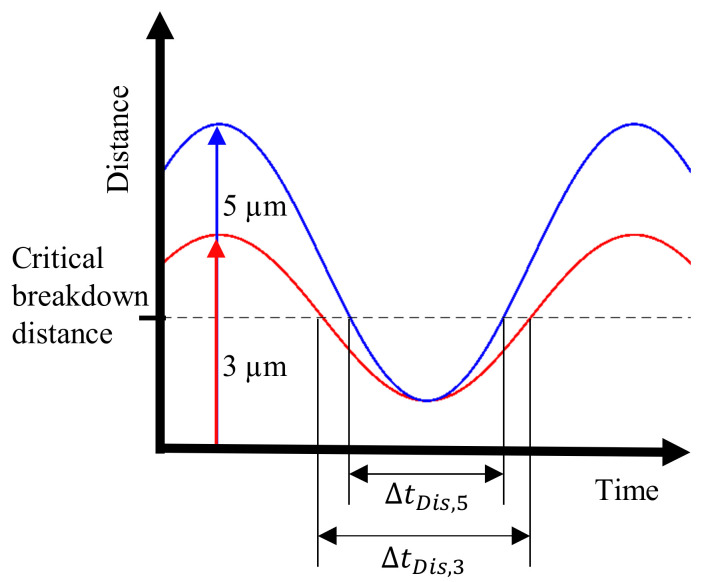
Distance of the tool electrode to the workpiece surface during one vibration cycle.

The tool follows a sinusoidal trajectory, and increasing frequency and amplitude of the vibration leads to a higher electrode velocity. As shown in Figure 13, the residence time of the tool in the beneficial discharge range decreases when the amplitude is changed from 3 µm to 5 µm.

### 3.4. Effect of Vibration on the Machining Time

**Figure 14:**
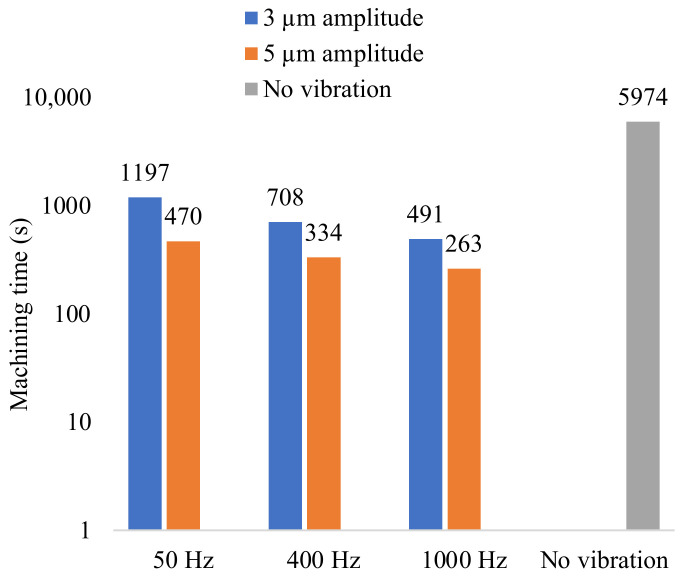
Machining time for a depth of 80 µm with the parameters specified in Table 2.

**Figure 15:**
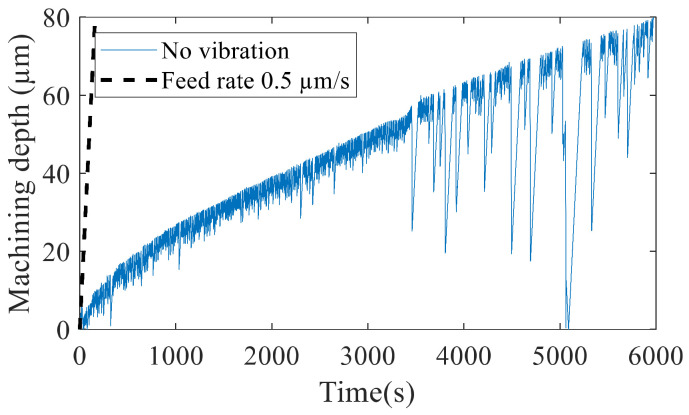
Tool feed without vibration and the parameters specified in Table 2.

The progression of the tool was recorded, and the machining time to a depth of 80 µm is illustrated in Figure 14. The highest machining time was recorded without vibration. As seen from the tool feed in Figure 15, the tool progression followed the specified feed rate of 5 µm/second for only about 5 µm and then slowed down significantly. The machine control performed a reciprocation motion of the tool after abnormal discharge was detected to flush debris from the gap. This jump motion occurred frequently, indicating bad flushing conditions and regular short-circuiting.

**Figure 16:**
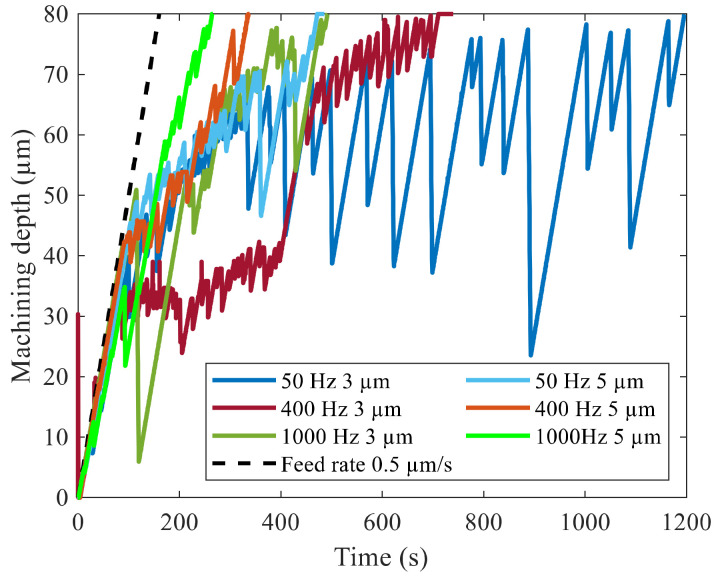
Tool feed with vibration and the parameters specified in Table 2.

The tool electrode feed with vibration over the machining time is illustrated in Figure 16. At a vibration with 3 µm amplitude, the machining rate followed the feed rate to a higher machining depth than without vibration. Tool jump motion did not occur up to a machining depth of 30 µm at 50 Hz and 400 Hz and up to 40 µm at 1000 Hz. The machining rate progressed slower from that point on. The frequency of tool reciprocation increased with a higher machining depth but remained less frequent than without vibration, as seen in Figure 15.

The range of stable machining was further increased at a vibration amplitude of 5 µm. Increasing tool jump motion was observed after a machining depth of 40 µm at a vibration frequency of 50 Hz and 400 Hz. At 1000 Hz, tool reciprocation became more frequent after a machining depth of 55 µm was reached. The jump motion was less frequent than at 3 µm machining. The beneficial effect of tool vibration on the machining time is therefore attributed to better machining conditions that reduce the frequency of tool retraction.

### 3.5. Effect of Vibration on Surface Roughness

**Figure 17:**
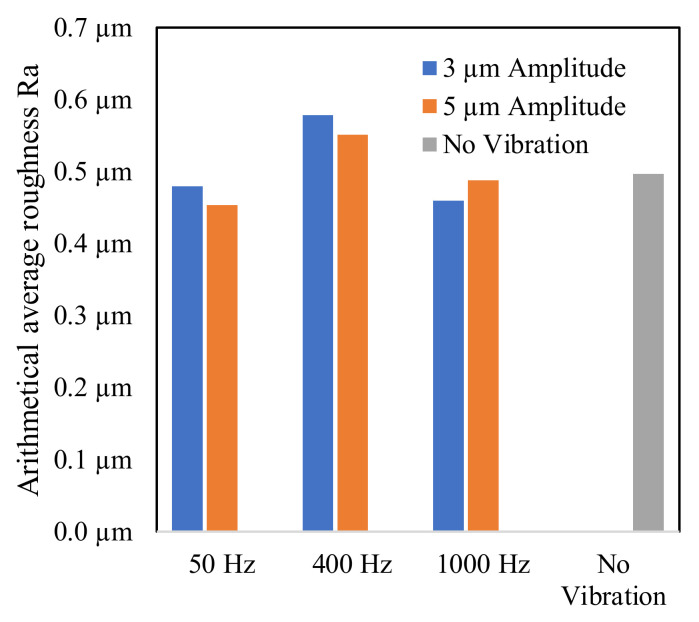
Average roughness of the array cavities.

The arithmetic average roughness, Ra, of the array cavities was measured. Due to the small size of the cavities, a non-standardized measurement was performed. A scanning pitch of 0.5 µm, a sampling pitch of 0.5 µm, and a cutoff length of 0.25 mm were used. The mean value of the nine cavities of each array was calculated. As illustrated in Figure 17, machining with vibration at 50 Hz and 1000 Hz led to similar surface roughness to that without vibration. The surface roughness was slightly higher at 400 Hz vibration. In addition, as a general trend, a higher vibration amplitude decreased the surface roughness, except at 1000 Hz frequency.

**Figure 18:**
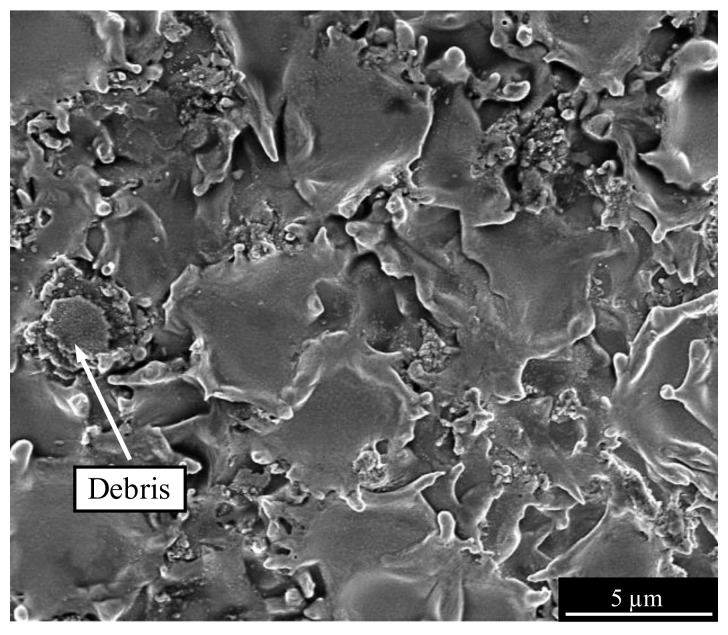
SEM image of the workpiece surface machined without vibration and the parameters specified in Table 2.

Figure 18 shows an SEM image of the workpiece surface machined without vibration. The surface is characterized by large flat craters without distinctive crater walls. The density of the discharge craters was low, and adhesion of debris particles was visible.

**Figure 19:**
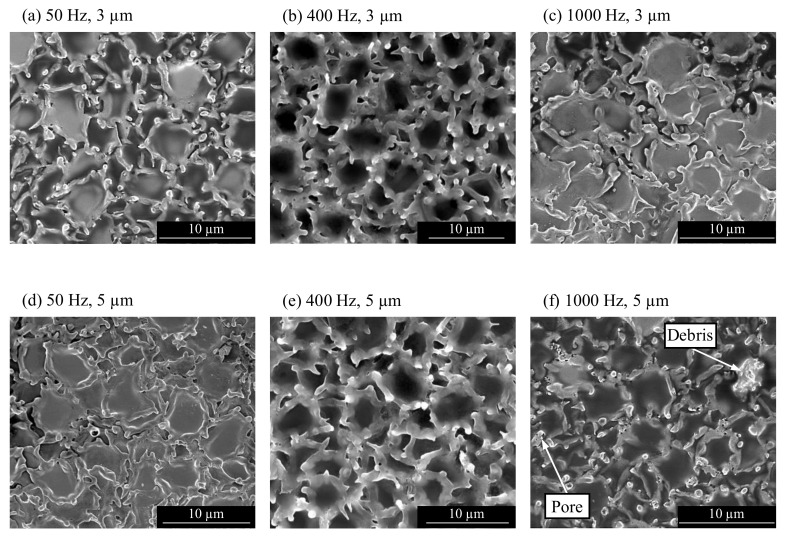
SEM image of the workpiece surface with the parameters specified in Table 2. The vibration frequencies and amplitudes are: (**a**) 50 Hz, 3 µm (**b**) 50 Hz, 5 µm (**c**) 400 Hz, 3 µm (**d**) 400 Hz, 5 µm (**e**) 1000 Hz, 3 µm (**f**) 1000 Hz, 5 µm.

The SEM images in Figure 19 show the sample surfaces machined with tool vibration. Compared to Figure 18, the density of the discharge craters is higher, and the discharge craters are smaller but deeper. The SEM observation of the sample surface in Figure 19 revealed differences in the crater size and shape with different vibration parameters. At 50 Hz and 1000 Hz, the discharge craters appeared flat with thin walls. The surfaces machined at 400 Hz frequency in Figure 19c,d showed deeper craters. The thickness of the crater walls was higher, with more remolten material on the crater edges. The sample machined at 1000 Hz and 5 µm in Figure 19f also showed a few adhered debris particles and porosity of the crater walls. This might have caused the higher roughness despite smaller crater depths compared to machining with vibration at 1000 Hz and 3 µm.

The observation of the workpiece surface and measurement of surface roughness indicated that the discharge craters changed with different vibration settings. As Wang et al. [33] reported, a higher amplitude of the electrode vibration can lead to a shallower crater depth as a result of a shift in the discharge plasma origin. This might be a reason for the decrease in surface roughness from 3 µm to 5 µm vibration amplitudes at the frequencies of 50 Hz and 400 Hz. Another reason might be that the crater density at a larger amplitude is higher, which causes more overlaps among the craters, and in turn, lower roughness.

A higher discharge energy can melt more workpiece material, resulting in a correlation to the crater size [34]. The variance of the crater size indicates changing discharge energy at different vibration parameters. This could result from a difference in the gap width. A changing gap width is also demonstrated by the increasing intensity of the discharges at higher frequencies. With a smaller gap width and high discharge intensity, the risk of debris adhesion is increased, which was observed on the workpiece machined at 1000 Hz and 5 µm vibration. The workpiece machined without vibration provides evidence of a high concentration of debris in the gap, resulting in low discharge energy with small craters and a high probability of debris adhesion.

### 3.6. Effect of Vibration on Hole Diameter Uniformity

One of the major challenges in micro-EDM of arrays is the non-uniformity of the machined features. Non-uniformity of the hole diameter was observed in experiments with and without tool vibration. So far, studies have focused on tool vibration in the ultrasonic range [12,20].

An experiment was performed with the same materials and parameters as the previous experiment specified in Table 2, and the hole diameter uniformity was studied. To prevent small deviations of the electrode diameter from influencing the results, the diametral overcut was used to assess the hole uniformity. The diametral overcut ratio is calculated according to Equation (2), where *d*_hole_ is the hole entrance diameter and *d*_electrode_ is the electrode diameter. The hole entrance area, *A*_hole_, and cross-sectional electrode area, *A*_electrode_, were evaluated by 3D laser scanning microscope (LEXT OLS4100, Olympus Corp., Tokyo, Japan).

(2) $$\mathrm{Overcut}\;\mathrm{Ratio}=\frac{\frac{\pi }{4}\;\left(d_{\mathrm{hole}}{}^{2}{-\;d}_{\mathrm{electrode}}{}^{2}\right)}{\frac{\pi }{4}{\;d}_{\mathrm{hole}}{}^{2}}=\frac{A_{\mathrm{hole}}{-\;A}_{\mathrm{electrode}}}{A_{\mathrm{hole}}}$$

**Figure 20:**
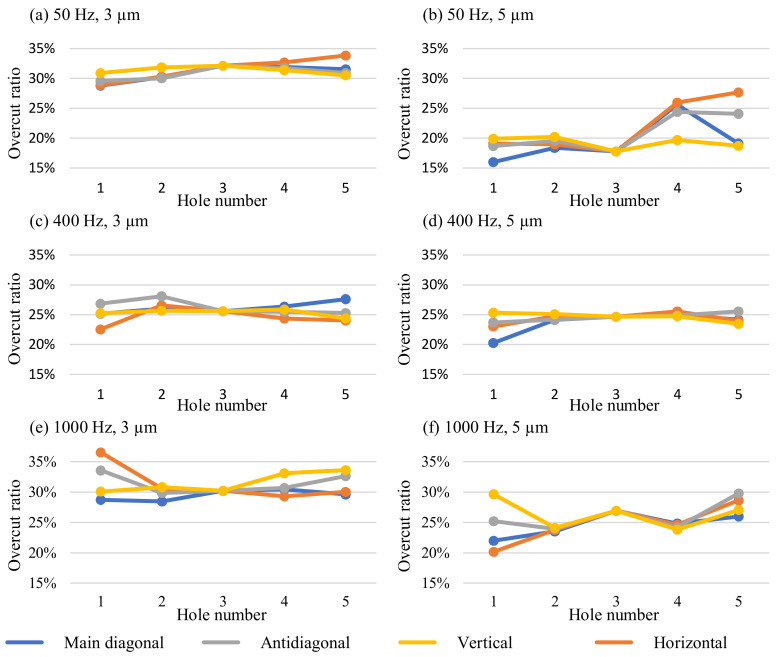
Ratio of the overcut to the hole entrance area with the parameters specified in Table 2. The vibration frequencies and amplitudes are: (**a**) 50 Hz, 3 µm (**b**) 50 Hz, 5 µm (**c**) 400 Hz, 3 µm (**d**) 400 Hz, 5 µm (**e**) 1000 Hz, 3 µm (**f**) 1000 Hz, 5 µm.

Figure 20 illustrates the overcut of the arrays. The evaluation was performed along the array’s main diagonal, antidiagonal, horizontal, and vertical directions. It must be noted that the variance of hole number 3 is zero, as it represents the central hole along with all evaluation directions.

As illustrated in Figure 20a, the array machined at 50 Hz and 3 µm showed a higher overcut in the center of the array, which matched the results of previous studies. A standard deviation of 1.31% from the mean overcut ratio of 30.95% was obtained. The overcut ratio at 50 Hz and 5 µm was smaller, with a mean value of 21.31%. Linked to the decreased overcut ratio is a smaller gap width. This might explain the non-uniform overcut seen in Figure 20b as a cause of a more difficult debris removal through the smaller side gaps. The standard deviation of the overcut ratio was 3.47%. Machining at 400 Hz and 3 µm resulted in a mean overcut ratio of 25.48%. The values for the individual holes of the array machined at 400 Hz and 3 µm are illustrated in Figure 20c. The irregularity of the overcut ratio was smaller than at 50 Hz and 3 µm vibration, as indicated by the standard deviation of 1.2%. At 400 Hz and 5 µm, the best uniformity of the hole overcut ratio was achieved with a standard deviation of 1.09%. As demonstrated by Figure 20d, a uniform overcut in the center of the array and deviations with smaller overcut at the edges were observed. The mean overcut ratio was 24.23%. Figure 20e shows the overcut at 1000 Hz and 3 µm. There was a high variance of the overcut ratio at the edges of the array, while the uniformity increased towards the center. This was also represented by the standard deviation of 2.13%. The mean overcut ratio of 31.28% indicated a higher gap width. A similar trend was observed at 1000 Hz and 5 µm, as detailed in Figure 20f. A scattered overcut ratio on the edges was recorded that caused a high standard deviation of 2.74%. As seen by the higher overcut ratio, the gap width increased towards the center. The variance of the overcut decreased likewise. A mean overcut ratio of 25.98% was obtained.

**Figure 21:**
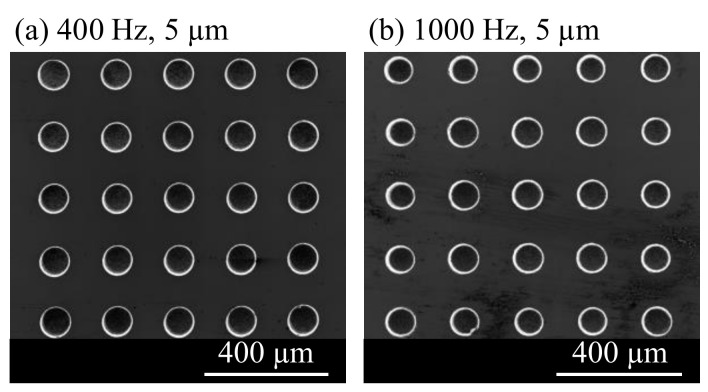
SEM images of 5 × 5 arrays (**a**) Machined with vibration at 400 Hz and 5 µm amplitude (**b**) Machined with vibration at 1000 Hz and 5 µm amplitude.

The higher overcut around the edges of the array was unexpected. One possible explanation would be that debris from the electrodes in the center is transported to the outside by the vibrating movement. The debris is sucked back in the holes at the edges of the array and leads to the increased debris concentration in these areas. In earlier findings, a larger hole diameter was attributed to bad debris flushing from the center holes of the array. Hole arrays machined at 400 Hz and 1000 Hz with 5 µm vibration amplitude are compared in Figure 21. A higher variance of the hole diameter can be seen in Figure 21b.

### 3.7. Effect of Vibration on Tool Wear

**Figure 22:**
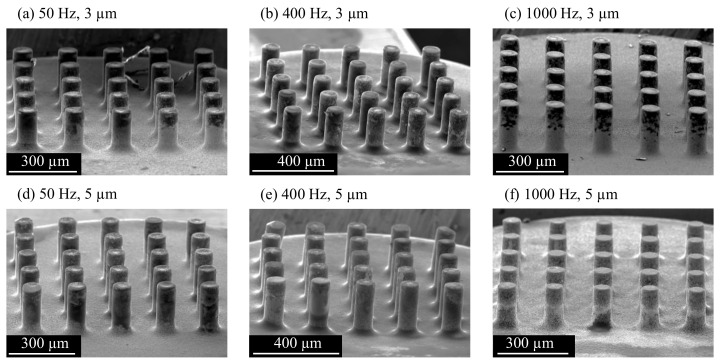
SEM images of the 5 × 5 array tools after use with the parameters specified in Table 2. The vibration frequencies and amplitudes are: (**a**) 50 Hz, 3 µm (**b**) 50 Hz, 5 µm (**c**) 400 Hz, 3 µm (**d**) 400 Hz, 5 µm (**e**) 1000 Hz, 3 µm (**f**) 1000 Hz, 5 µm.

The tool wear was studied by SEM microscopy after a single use of the tool. Compared to the tool before use in Figure 4b, the rounded edges of the array used at 50 Hz and 3 µm vibration in Figure 22a indicate significant wear. The tool wear also caused the formation of craters on the top surface of the electrodes. Deposition of carbon and debris can be seen as black material in the upper part of the electrodes.

The effects of wear were smaller on the array used at 400 Hz and 3 µm vibration. As shown in Figure 22b, craters were formed on some electrodes, mainly in the center of the array. A similar amount of carbon and debris was deposited on the electrode surface. The array used at 1000 Hz and 3 µm experienced less wear, as indicated by the sharper edges and flat electrode top faces in Figure 22c. Less material was deposited on the electrodes, leaving discharge craters visible. The tools used with a vibration amplitude of 5 µm showed a similar wear characteristic. High wear with craters in the electrode center was observed at 50 Hz vibration frequency in Figure 22d. Carbon and debris were deposited mainly on the electrode side faces, and the amount was smaller compared to the tool used at 50 Hz and 3 µm. The tool wear was significantly smaller after machining at 400 Hz and 5 µm, and no craters were formed on the electrodes, but a high build-up of material can be seen on the electrodes in Figure 22e. Vibration at 1000 Hz and 5 µm resulted in minimal tool wear. The edges of the electrodes were only slightly rounded, and the top surface remained flat, as shown in Figure 22f. Very little debris and carbon was deposited on the side faces, but a layer of material formed on the top surfaces of the electrodes.

### 3.8. Mechanism of Vibration-Induced Discharge Intensity Change

Tool vibration caused regular discharge intervals, which is in line with the concept of a beneficial discharge range. It was proposed that discharges only occur when the distance between tool and workpiece electrode is below a certain threshold distance [6]. Tong et al. [6] suggested a relationship between the optimum amplitude and the discharge distance between the tool and the workpiece. However, the model considered vibration to reduce the negative influence of a low response frequency of the machine control. It failed to address the effect of vibration on the discharge process.

**Figure 23:**
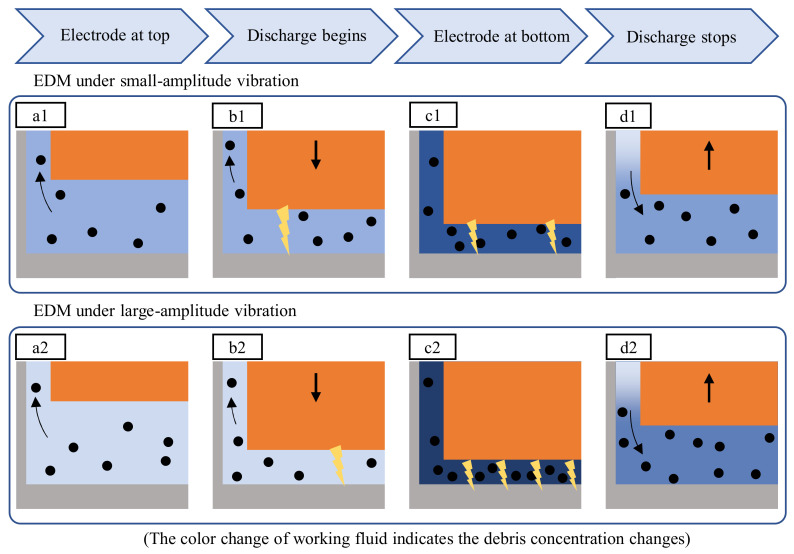
Mechanism of the increase of the discharge intensity with a higher vibration amplitude. (**a1**) Electrode at top, (**b1**) discharge begins, (**c1**) electrode at bottom, and (**d1**) discharge stops under small-amplitude vibration; (**a2**) Electrode at top, (**b2**) discharge begins, (**c2**) electrode at bottom, and (**d2**) discharge stops under large-amplitude vibration.

It was observed in this study that the discharge intensity increased with higher amplitudes. Figure 23 compares the interaction of debris and discharges over one vibration cycle at low and high amplitude vibration. Due to the flushing of debris formed in the previous vibration cycle, the debris concentration in the gap is low when the electrode is in the top position, as shown in Figure 23(a1,a2). It is assumed that more effective flushing at higher amplitudes contributes to a lower debris concentration in Figure 23(a2). Therefore, the dielectric breakdown strength is higher. The first discharge of the cycle at the higher amplitude in Figure 23(b2) occurs at a smaller gap width, compared to the lower amplitude seen in Figure 23(b1). This discharge forms new debris particles in the gap, and the debris concentration increases. The rising debris concentration leads to a reduced breakdown strength and facilitates more discharges. Due to the smaller fluid volume at the higher amplitude, the debris concentration in Figure 23(c2) is significantly higher than in Figure 23(c1). More discharges are triggered as a result of the drastically reduced breakdown strength. As these discharges also release new debris particles, a self-reproduction effect of the discharges is achieved. This situation promotes the overlapping of craters, leading to a decrease in surface roughness, as seen in Figure 17 and Figure 19. When the tool electrode moves upwards, new fluid enters the gap and reduces the debris concentration. The discharge process stops when the tool electrode surpasses the critical breakdown distance to the workpiece, as seen from Figure 23(d1,d2).

The concentrated discharge phenomenon might occur when the debris concentration becomes extremely high towards the end of the regular discharge period. As a consequence of the very low breakdown strength, the concentrated discharge can continue while the regular discharge is stopped. The gap observation showed that the discharge concentration was more likely to subside with tool vibration before arcing could occur. This provides evidence that the discharge period is linked to the material removal rate, as the material removal rate benefits from a high discharge rate and low frequency of short-circuiting. The tool vibration parameters must be carefully selected to achieve the optimum machining conditions. If the frequency is too low, the discharge period will continue too long, and short-circuiting will occur. If the frequency is too high, fewer discharges per cycle will occur, and the machining efficiency is reduced.

## 4. Conclusions

The effect of tool vibration on the electrical discharge machining process with micro array electrodes was studied by direct observation of the discharge gap. The influence of vibration on the microscopic surface topography and array uniformity was further evaluated. The following conclusions result from the study:

1. The uniformity of the discharge spot distribution on the individual electrodes of the array was improved by tool vibration. The higher uniformity resulted from a higher discharge rate and fewer irregular discharges, such as discharge concentration and short-circuiting.
2. Tool vibration led to a periodic discharge process. The discharges only occurred when the tool electrode was at a certain distance to the workpiece due to the electrical breakdown strength of the fluid.
3. Higher vibration frequency and amplitude reduced the ratio of the discharge period to the time without discharges in the vibration cycles. With increasing vibration amplitude, the intensity of the discharge period increased.
4. Tool vibration reduced the machining time. An increase in both vibration frequency and amplitude improved the machining time.
5. Vibration assistance led to a change in the surface roughness of the machined surfaces. This was primarily due to a difference in the discharge crater size and resolidified material. The crater size and density were reduced without vibration, and more debris adhered to the surface.
6. Arrays of 25 holes were machined, and the hole diameter uniformity was studied. The best uniformity was achieved at a vibration frequency of 400 Hz.

This work has demonstrated the potential of using tool vibration for assisting micro EDM with array electrodes and other complex geometries. The gap observation clarified the phenomena of the periodic discharge, which is necessary for obtaining the optimal vibration parameters. Vibration frequency and amplitude influenced the sample roughness and hole diameter uniformity. Further research will include the investigation of discharge energy and the direct observation of debris in the gap, among other things.

## Figures and Tables

**Table 1:** Vibration parameters selected for the experiments.

Frequency	Axial Amplitude	Radial Amplitude (x/y)
(Hz)	(µm)	(µm/µm)
50	3	0.12/0.11
	5	0.24/0.2
400	3	0.58/0.58
	5	1.05/1.05
1000	3	0.94/0.81
	5	1.1/0.63

**Table 2:** Experimental parameters of micro EDM experiments.

Parameter	Value
Voltage (V)	100
Capacitance (pF)	220
Feed rate (µm/s)	5
Machining depth (µm)	80
Workpiece electrode	Anode: 4H-SiC (+)
Tool electrode	Cathode: Cu (−)
Working fluid	EDM oil (CASTY-LUBE EDS)

**Table 3:** High-speed camera settings.

Parameter	Value
Framerate (fps)	20,000
Recording time (s)	8.7345
Resolution	256 × 256 pixel

**Table 4:** Average intensity of discharge during the discharge periods.

Frequency	Number of Discharges Per ms	
(Hz)	Amplitude 3 µm	Amplitude 5 µm
50	100	123
400	63	80
1000	76	115

## Data Availability

Not applicable.
